# Value of syndromic surveillance within the Armed Forces for early warning during a dengue fever outbreak in French Guiana in 2006

**DOI:** 10.1186/1472-6947-8-29

**Published:** 2008-07-02

**Authors:** Jean-Baptiste Meynard, Hervé Chaudet, Gaetan Texier, Vanessa Ardillon, Françoise Ravachol, Xavier Deparis, Henry Jefferson, Philippe Dussart, Jacques Morvan, Jean-Paul Boutin

**Affiliations:** 1 Institut Pasteur de la Guyane, Cayenne 97306, French Guiana; 2 Université de la Méditerranée, Marseille 13385, France; 3 Institut de Médecine Tropicale du Service de santé des armées, Marseille 13998, France; 4 Cellule Inter Régionale d'Epidémiologie Antilles-Guyane, 97306, French Guiana; 5 Direction de la Santé et du Développement Social de la Guyane, 97306, French Guiana; 6 Ecole du Val-de-Grâce, Paris 75230, France; 7 Liverpool School of Tropical Medicine, Liverpool L3 5QA, UK

## Abstract

**Background:**

A dengue fever outbreak occured in French Guiana in 2006. The objectives were to study the value of a syndromic surveillance system set up within the armed forces, compared to the traditional clinical surveillance system during this outbreak, to highlight issues involved in comparing military and civilian surveillance systems and to discuss the interest of syndromic surveillance for public health response.

**Methods:**

Military syndromic surveillance allows the surveillance of suspected dengue fever cases among the 3,000 armed forces personnel. Within the same population, clinical surveillance uses several definition criteria for dengue fever cases, depending on the epidemiological situation. Civilian laboratory surveillance allows the surveillance of biologically confirmed cases, within the 200,000 inhabitants.

**Results:**

It was shown that syndromic surveillance detected the dengue fever outbreak several weeks before clinical surveillance, allowing quick and effective enhancement of vector control within the armed forces. Syndromic surveillance was also found to have detected the outbreak before civilian laboratory surveillance.

**Conclusion:**

Military syndromic surveillance allowed an early warning for this outbreak to be issued, enabling a quicker public health response by the armed forces. Civilian surveillance system has since introduced syndromic surveillance as part of its surveillance strategy. This should enable quicker public health responses in the future.

## Background

One of the main objectives of health surveillance systems is to provide early warning of disease outbreaks, which allows for acceleration and optimization of a public health response. In the current context of international bioterrorism threats, early warning has become increasingly important, and many countries, including the Armed Forces based in French Guiana, have incorporated warning systems into their civilian and military surveillance systems [1,2].

French Guiana is a French overseas department in South America where tropical diseases responsible for outbreaks exist, such as dengue fever. Dengue fever is a viral disease, caused by an arbovirus of the *Flaviviridae *family in the *Flavivirus *genus. There are four viral serotypes of this virus, designated DENV-1, DENV-2, DENV-3 and DENV-4. It is transmitted by a mosquito vector called *Stegomyia aegypti *(formerly *Aedes aegypti*). Dengue is the predominant arthropod borne viral disease affecting humans [3]. Around the world, 2.5 billion people in more than 100 countries are exposed to this virus annually. There are 50 to 100 million of cases per year, with 500,000 hospitalizations and 22,000 deaths annually worldwide [4]. The World Health Organization has estimated that there has been a 30-fold increase in the incidence of dengue in the past 50 years [4]. There is no vaccine and no curative treatment available. The only operational public health strategy of defense is prevention through vector control [5].

Until 2006, the surveillance of dengue fever in French Guiana was based on the weekly surveillance of biologically confirmed cases within the 200,000 general population of French Guiana (table 1). The definition criteria were: virus isolation on mosquito cells, viral RNA detection by reverse transcription-PCR (RT-PCR), or a serological test based on immunoglobulin M (IgM)-capture enzyme-linked immunosorbent assay (MAC-ELISA) [6]. The biological laboratories sent weekly results to the CVS ("Cellule de Veille Sanitaire") of French Guiana, working in collaboration with CIRE ("Cellule Inter Regionale d'Epidemiologie") of French Guiana and French West Indies.

**Table 1:** General presentation of 2SE FAG (syndromic surveillance), SEA (clinical surveillance) and CVS (biological surveillance) systems.

	**2SE FAG**	**SEA**	**CVS**
**Date of first use**	18/10/2004	01/01/1994	01/01/1995
**Population coverage and main characteristics of the population**	3 000 military people 11,8% women 88,2% men average age = 34 years old	3 000 military people (id 2SE FAG) 11,8% women 88,2% men average age = 34 years old	200 000 people (general population) 50,5% women 49,5% men <15 years old: 35,4% >65 years old: 3,8%
**Heath provision**	15 general practitioners (GPs)	15 GPs (id 2SE FAG)	3 public hospitals 24 health centres 70 GPs 7 biological laboratories (4 private and 3 public)
**Type of disease definition criteria**	Syndromic	1. Clinical and biological (inter-epidemic period)	Biological
		2. Clinical (epidemic period) 3.	
**Timing of reports**	Real time	Weekly	Weekly
Statistical analysis *	CPEG, EWMA	CPEG	Empirical method
**Periodicity of feed-back**	Real time	Weekly	Monthly

* CPEG: Current Past Experienced Graph

EWMA: Exponentiel Weighted Moving Average

For the Armed Forces based in French Guiana, the surveillance of dengue fever is based on the traditional clinical military mandatory system SEA ("Surveillance Epidemiologique dans les Armees"), functioning by the weekly surveillance of 63 health events within the 3,000 soldiers in French Guiana (table 1). Dengue fever is one of these diseases. Its definition criteria are different, depending on the epidemiological context. During an interepidemic period, a dengue fever case is defined by a clinical picture (fever, headache and at least 2 symptoms among retro-orbital pains, myalgia, arthralgia, cutaneous rash, with or without hemorrhagic symptoms) and a biological confirmation: virus isolation, viral RNA detection by reverse transcription-PCR (RT-PCR), or a serological test based on immunoglobulin M (IgM). During an epidemic period, only the clinical picture is necessary. The cases are recorded by the military general practitioners (GPs) and weekly sent to the DIASS ("Direction Interarmées du Service de santé") in Cayenne and to the IMTSSA ("Institut de Médecine Tropicale du Service de santé des armées") in Marseilles.

To enhance the performance of this clinical surveillance, the solution was to create a new system: the 2SE FAG system («Surveillance Spatiale des Epidémies au sein des Forces Armées en Guyane»). This prototype was set up in October 2004 [7,8], combined with the clinical system. Its main objectives are to allow operational study of a real-time surveillance system using, to evaluate the value of such a system compared to the traditional surveillance and to identify interoperability criteria for allied cooperation (countries of North-Atlantic Treaty Organization). It is a real time surveillance system of fever within the 3,000 service men of French Guiana (table 1). A case of dengue fever is defined as a sudden onset of fever (equal or more than 38 degrees Celsius), with no evidence of other infection (particularly malaria, with rapid diagnostic test and/or thick blood smear negative), associated with one or more non specific symptoms including headache, myalgia, arthralgia and/or retro-orbital pains. These suspected cases are recorded by military GPs and nurses (the same people as for the clinical surveillance) and sent in real time to the several servers set up in the epidemiological unit of IPG ("Institut Pasteur de la Guyane") in Cayenne, in the DIASS of Cayenne and in the IMTSSA in Marseilles.

During the first quarter of 2006 French Guiana suffered the largest dengue outbreak in its history. The virus was first detected in the west of the country and quickly spread across the whole country. This outbreak was detected by both military and civilian surveillance systems and specific public health responses were been implemented by military and civilian health authorities.

The main objective of this survey was to study the value of the syndromic surveillance system set up within the armed forces for early warning, compared to the military traditional clinical system during the 2005–2006 outbreak of dengue fever in French Guiana. The other objectives were to highlight issues involved in comparing military and civilian surveillance systems and to discuss the interest of syndromic surveillance for public health response.

## Methods

Until 2006, the analysis of the civilian surveillance data was performed by CVS in Cayenne, using an empiric method, which had an alarm threshold of 6 cases per week. If the number of cases exceeded this threshold for 2 consecutive weeks, further investigations were carried out.

For the analysis of military clinical surveillance data (SEA), a weekly statistical non automated analysis is performed by IMTSSA in Marseilles, using the Current Past Experienced Graph [9] (CPEG). This method permits comparison of the observed number of cases with historical data, generated from the past 3–5 years, using Student and/or Poisson statistical laws. Those laws both give an answer to the question "knowing the average number of expected events during a period of time, what is the probability to observe the current situation?" The Poisson law is usable for rare events (when it is not possible to use binomial law) and Student law is derived from the normal law.

The statistical analysis of military syndromic surveillance data (2SE FAG) is automated and uses both the CPEG [9] and the Exponential Weighted Moving Average (EWMA) [10], which is a control chart method permitting to smooth the curves of temporal data. These analyses are performed within the 2SE FAG analysis network, called CS^3 ^("communauté de services Internet pour la surveillance syndromique"), and allows continuous definition of the epidemiological situation.

To reach the first objective of comparing syndromic and clinical military surveillance systems, two statistical methods were used to carry out the retrospective analysis of data. The main studied performance was the early warning capacity. Therefore, the choice was to use practical and robust statistical methods, well known to generate some signals and to launch some alarms in routine use [11,12]. The first chosen method was the CPEG [9], commonly used by both military systems and currently coded 0 if the observed data weren't outside the historical limits ("normal" situation), + if the observed data were outside the historical limits by more than 2 standard deviations ("pre-alarm") and ++ if by more than 3 standard deviations compared to the expected data ("alarm"). The second method used was that of cumulated amounts (CUSUM) [13,14], non routinely used by the systems in French Guiana and used with a verification aim. This method, commonly used for quality control in industry, has been adapted to epidemiological surveillance, and works using incidence and incidence rate. It is a control chart method, a tool to determine whether a manufacturing or business process is in a state of statistical control or not [15]. If the chart indicates that the process being monitored is not in control, the pattern it reveals can help determine the source of variation to be eliminated to bring the process back into control. In our study, if the curve goes up, there is an increasing of incidence or incidence rate compared to the reference (expected data for the same period); if it lowers, there is a fall compared to the reference; if the curve is parallel with the abscissa axis, then incidence or incidence rate is stable.

To implement those methods and allow comparison between systems, it was necessary to construct weekly data for syndromic surveillance, which collects data in real time, i.e. several times a day. Incidences and incidence rates were then calculated. It was also necessary to have at least 3 years of historical data for each system. This was the case for clinical surveillance, but not for syndromic surveillance, which was set up in October 2004 and effectively operational in August 2005. Because of this, the historical data base was constructed from the clinical surveillance data, including the whole health events having fever in their clinical picture (8 health events).

Both statistical methods also permitted to realize a retrospective analysis of laboratory surveillance data. Military and civilian authorities gave information about public health response (time, nature, graduation).

The statistical analysis was carried out with EPI_db^®^, version 1.0 (Société Sarvis, Kourou, French Guiana).

The study period was between week 41 of 2005 and week 25 of 2006. All the surveillance data were anonymous and recorded in the same secure data base, after the agreement of military and civilian public health authorities and the approval of the French "commission nationale de l'informatique et des libertés" (N°1149659) [see Additional file 1].

## Results

**Figure 1:**
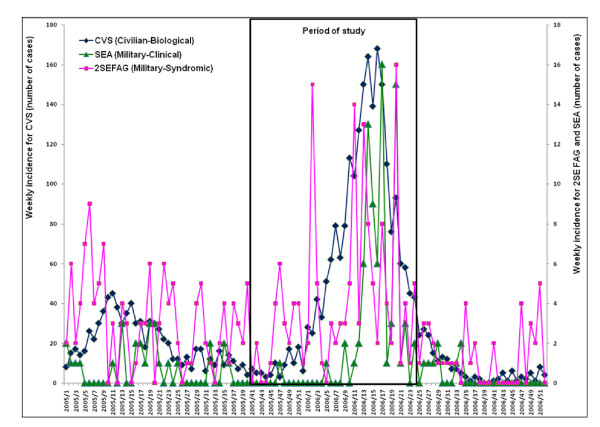
Weekly case counts of dengue fever (military clinical surveillance – SEA) and of suspected dengue fever cases (military syndromic surveillance – 2SE FAG) within the armed forces in French Guiana, and weekly case counts of biologically confirmed cases (civilian biological surveillance – CVS) within the general population in 2005 and 2006 (period of study between week 41 of 2005 and week 25 of 2006).

**Figure 2:**
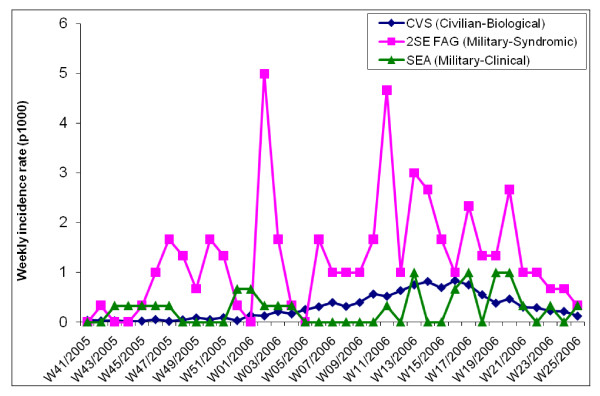
Weekly incidence rates (number of cases per 1000 people) of dengue fever (clinical surveillance – SEA) and of suspected dengue fever cases (syndromic surveillance – 2SE FAG) within the armed forces in French Guiana, and weekly incidence rate of biologically confirmed cases (laboratory surveillance – CVS) within the general population, from week 41 of 2005 to week 25 of 2006.

At the beginning of the study period, in October 2005, the epidemiological situation of dengue fever in French Guiana was normal, after the end of a dengue fever outbreak (serotype DENV-3) which had occurred during the previous months (from March to September 2005). Incidence and incidence rates of dengue fever at this time were considered to be normal. Figures 1 and 2 show weekly incidence and incidence rates of the 3 systems, syndromic and clinical surveillance reporting on the armed forces and laboratory surveillance reporting on the civilian population. It was difficult to precisely evaluate the beginning of the outbreak. Figure 1 shows the size of the outbreak recorded by all systems. Figure 2 shows that the first increases of incidence rate were detected by syndromic surveillance. This figure indicates that the mechanisms of spread were different within the military and civilian populations. Biological analysis showed that the circulating serotype during this outbreak was the DENV-2 serotype.

Tables 2 and 3 show CPEG results for both syndromic and clinical surveillance. Syndromic surveillance detected an abnormal incidence of dengue fever in week 43, and again during week 44, 3 to 4 weeks before the other systems. During the outbreak, the results oscillated between normal and abnormal situations within the military population with consistent gaps of several weeks between syndromic and clinical surveillance. Among the general population, the test showed an abnormal dengue fever incidence for several months, as shown in figure 1.

**Table 2:** Results of CPEG tests realized for 2SE FAG (syndromic surveillance), SEA (clinical surveillance) and CVS (biological surveillance) systems, with Student and Poisson statistical laws, from observed incidences of the week 41 of 2005 to the week 25 of 2006

**Week**	**2SE FAG (Military-Syndromic)**		**SEA (Military-Clinical)**		**CVS (Civilian-Biological)**	
	
	**Student**	**Poisson**	**Student**	**Poisson**	**Student**	**Poisson**
W41/2005	0	0	0	0	0	0
W42/2005	0	0	0	0	0	0
W43/2005	0	**+**	0	0	0	0
W44/2005	**+**	**++**	0	0	0	0
W45/2005	**++**	**++**	0	0	0	0
W46/2005	**+**	**++**	**+**	**++**	0	**+**
W47/2005	0	0	**++**	**++**	0	0
W48/2005	0	0	**++**	**++**	0	0
W49/2005	0	**+**	0	0	**+**	**++**
W50/2005	**++**	**+**	0	0	**+**	**++**
W51/2005	**++**	**+**	0	0	**++**	**++**
W52/2005	**+**	0	0	0	**++**	**++**
W01/2006	0	0	**++**	**++**	**++**	**++**
W02/2006	**++**	**++**	**++**	**++**	**++**	**++**
W03/2006	**++**	**++**	**++**	**++**	**++**	**++**
W04/2006	**++**	**++**	**++**	**++**	**++**	**++**
W05/2006	**++**	**++**	**++**	**+**	**++**	**++**
W06/2006	0	0	0	0	**++**	**++**
W07/2006	0	0	0	0	**++**	**++**
W08/2006	0	0	0	0	**++**	**++**
W09/2006	0	0	0	0	**++**	**++**
W10/2006	0	0	0	0	**++**	**++**
W11/2006	**+**	**++**	0	0	**++**	**++**
W12/2006	0	**+**	0	0	**++**	**++**
W13/2006	**+**	**++**	**++**	**+**	**++**	**++**
W14/2006	**+**	**++**	**++**	**+**	**++**	**++**
W15/2006	0	0	0	0	**++**	**++**
W16/2006	0	0	**+**	**+**	**++**	**++**
W17/2006	0	0	**+**	**+**	**++**	**++**
W18/2006	0	0	**+**	**+**	**++**	**++**
W19/2006	0	0	**++**	**++**	**++**	**++**
W20/2006	0	0	**++**	**++**	**++**	**++**
W21/2006	0	0	**++**	**++**	**++**	**++**
W22/2006	0	0	**++**	**++**	**++**	**++**
W23/2006	0	0	**+**	**+**	**++**	**++**
W24/2006	0	0	0	0	**++**	**++**
W25/2006	0	0	0	0	**++**	**++**

0: Weekly data included within historical limits

+: Weekly data beyond historical limits (more than 2 standard deviations)

++: Weekly data beyond historical limits (more than 3 standard deviations)

**Table 3:** Results of CPEG tests realized for 2SE FAG (syndromic surveillance), SEA (clinical surveillance) and CVS (biological surveillance) systems, with Student and Poisson statistical laws, from observed incidence rates of the week 41 of 2005 to the week 25 of 2006

**Week**	**2SE FAG (Military-Syndromic)**		**SEA (Military-Clinical)**		**CVS (Civilian-Biological)**	
	
	**Student**	**Poisson**	**Student**	**Poisson**	**Student**	**Poisson**
W41/2005	0	**+**	0	0	0	0
W42/2005	0	0	0	0	0	0
W43/2005	0	0	0	0	0	0
W44/2005	**++**	0	0	0	0	0
W45/2005	**+**	0	0	0	0	0
W46/2005	**+**	0	**+**	0	0	0
W47/2005	0	0	**++**	0	0	**+**
W48/2005	0	0	**++**	0	0	0
W49/2005	0	0	0	0	**+**	**++**
W50/2005	**++**	0	0	0	**+**	**+**
W51/2005	**++**	0	0	0	**++**	**++**
W52/2005	**+**	0	0	**+**	**++**	0
W01/2006	0	0	**++**	**+**	**++**	**++**
W02/2006	**++**	**++**	**++**	0	**++**	**++**
W03/2006	**++**	0	**++**	0	**++**	**++**
W04/2006	**++**	0	**++**	0	**++**	**++**
W05/2006	**++**	0	**++**	0	**++**	**++**
W06/2006	0	0	0	0	**++**	**++**
W07/2006	0	0	0	0	**++**	**++**
W08/2006	0	0	0	0	**++**	**++**
W09/2006	0	0	0	0	**++**	**++**
W10/2006	0	0	0	0	**++**	**++**
W11/2006	**+**	**++**	0	0	**++**	**++**
W12/2006	0	0	0	0	**++**	**++**
W13/2006	**+**	**+**	**++**	**++**	**++**	**++**
W14/2006	**+**	0	**++**	0	**++**	**++**
W15/2006	0	0	0	0	**++**	**++**
W16/2006	0	0	0	0	**++**	**++**
W17/2006	0	0	0	**+**	**++**	**++**
W18/2006	0	0	0	0	**++**	**++**
W19/2006	0	0	**++**	**+**	**++**	**++**
W20/2006	0	0	**++**	**+**	**++**	**++**
W21/2006	0	0	**++**	0	**++**	**++**
W22/2006	0	0	**++**	0	**++**	**++**
W23/2006	0	0	**+**	0	**++**	**++**
W24/2006	0	0	0	0	**++**	**++**
W25/2006	0	0	0	0	**++**	**++**

0: Weekly data included within historical limits

+: Weekly data beyond historical limits (more than 2 standard deviations)

++: Weekly data beyond historical limits (more than 3 standard deviations)

**Figure 3:**
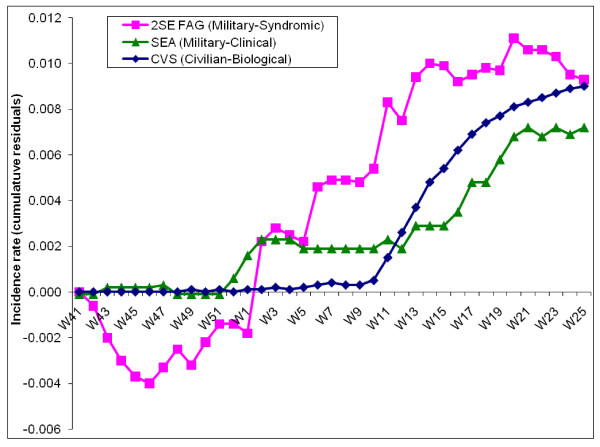
CUSUM for weekly incidence rates of dengue fever (military clinical surveillance – SEA) and of suspected dengue fever cases (military syndromic surveillance – 2SE FAG) within the armed forces in French Guiana, and CUSUM of weekly incidence rate of biologically confirmed cases (civilian biological surveillance – CVS) within the general population, from week 41 of 2005 to week 25 of 2006.

This result was confirmed by the CUSUM analysis, which gave results for incidences and incidence rates. The analysis of the incidence rates curve (figure 3) showed a gap of several weeks between the 3 systems, syndromic surveillance was the first to detect an abnormal signal during week 46. The timing of detection of an abnormal signal was not totally synchronous between the two statistical methods, but the gap between the 3 systems was shown by both methods.

In terms of public health response, the sanitary military authorities decided upon a reinforcement of collective and individual measures of vector control, based on the results provided by the automated analysis of syndromic surveillance. A pre-alarm was activated during week 41, this was not confirmed for three more weeks. The real alarm with the armed forces started during week 44 of 2005. The epidemiological investigation involved various steps commonly used to investigate syndromic surveillance signals [16]: confirmation of the existence of the outbreak, verification of the diagnosis, estimation of the number of cases, orientation to person, place and time, development and evaluation of a hypothesis, implementation of control measures and communication of findings. Following the confirmation of the outbreak, insecticides were used massively within the military camps, both inside buildings and outdoors all over the camps. All sites likely to harbor mosquito larvae were destroyed or treated. Service personnel stopped wearing short clothes and used repellents more often. Mosquito nets were used when sleeping whether during the day or night. Training for vector control was strengthened and specific information distributed extensively.

On the civilian side, vector control actions which had existed at the beginning of the dengue-2 outbreak were essentially an extension of those that had been implemented the previous summer due to the dengue-3 outbreak. The end of this outbreak had not been clearly announced and the local vector control teams were still active in the field. More time was necessary to detect the new dengue fever outbreak, in particular because CVS did not use any statistical tool to identify an increase in cases above a threshold. Several weeks were also necessary for the local vector borne disease committee to request a strengthening of existing vector control measures, with an involvement of the local population and its elected members. Specific training was set up within the communities and communication and awareness campaigns were organized among schools and among the general population.

The outbreak ceased in July 2006. The final toll was 149 suspected cases and 15 biologically confirmed cases among military personnel (attack rate = 5 cases per 1,000). On the civilian side, the final toll was 2,500 biologically confirmed cases (attack rate = 13 cases per 1,000). The civilian cases included 204 hospitalized patients with a confirmed dengue: 13% were dengue hemorrhagic fever cases, and 60% were scored as severe dengue cases without haemorrhage. Moreover, 4 deaths associated with confirmed dengue were reported (3 children and 1 adult).

## Discussion

This survey has carried out its main objective, showing that syndromic surveillance allowed an early detection of the dengue fever outbreak which had occurred in French Guiana during the first quarter of 2006, before the reference and mandatory clinical military system. With all the statistical methods used, syndromic surveillance detected an abnormal situation several weeks before clinical surveillance. There were no existing statistical methods to determine if those dates were significantly different from each other, contrary to simulated outbreaks [12]. This earlier warning allowed a quicker public health response by the armed forces. During the outbreak, several other alarms occurred, with a lag of time between the systems, showing that the outbreak was spreading to other military units and that specific public health measures had to be reinforced, especially vector control.

Other statistical methods could have been used to compare performances of the systems, as proposed in different studies [11,12]. The used methods were chosen to allow study of the systems' early warning capacity, especially timeliness. Some recent studies have underlined the interest of CUSUM, which for example performed significantly better than the methods of early aberration reporting system (EARS) across all the scenarios evaluated [17]. Other methods have been used in a separate survey to evaluate the other characteristics and parameters of both systems [18].

The analysis of syndromic surveillance used historical data prior to the creation of the system, using clinical recorded events of fever, not just dengue fever. This has certainly introduced a bias of classification into the study, which was difficult to control.

The outbreak had an important impact upon the military population, but the attack rate was far lower than within the civilian population. It was not possible to determine whether early warning and response resulted in avoidance of any cases. It was therefore impossible to calculate the number of avoided cases when using syndromic surveillance and to quantify the real benefit compared to clinical surveillance.

This study has also provided some elements of comparison between two different surveillance systems, civilian and military. But it was extremely difficult, indeed even impossible, to rigorously compare systems which don't use the same diseases definition criteria for surveillance within populations which were quite different in regard to their exposures and access to medical care. Even if the outbreak has followed a different time course within the two populations, those results have been presented anyhow, because it seemed more interesting to present what was known for those two intermingling populations than to provide only military surveillance data, and because military and civilian public health authorities worked very closely. Statistical analysis showed that syndromic surveillance was able to detect an abnormal signal before laboratory surveillance, as CVS didn't use any statistical tool at this time. Also, laboratory surveillance was only based on the surveillance of confirmed cases, in a French overseas department where logistical problems represent a restrictive factor for the dispatch of biological samples to laboratories. It was clear that cases reported by laboratory surveillance were not totally representative of the real situation of dengue fever within the general population. It is one of the reasons why the health authorities decided during the outbreak, in April 2006, to reinforce the laboratory surveillance system by introducing syndromic surveillance. These two types of surveillance are complementary; both contribute different surveillance data and together allow a better assessment of the epidemiological situation and therefore a better public health response. This new civilian system took into account the experience of other systems' [19], which have underlined the importance of surveillance system quality, the integration of syndromic surveillance with public health response and the importance of guidelines for informaticians, public health managers and general practitioners potentially involved. The addition of syndromic surveillance required the involvement of numerous new contributors in French Guiana (a network of GPs, health centres, hospital emergency units, hospital wards and the armed forces) and a new coordination team. It allowed an estimation of the impact of the 2006 outbreak, recording 16,200 suspected cases whereas the previous system counted only 2,500 confirmed cases.

On both military and civilian sides, that experience shows that a strategy based only on biological results gives incomplete data and the addition of a syndromic surveillance system gives more information. However, syndromic surveillance is associated with an increased risk of false alarms and of system saturation in case of outbreak. The use of syndromic surveillance has been validated previously, for example the exploitation of respiratory data [20]. However, some studies suggest that syndromic surveillance systems are unlikely to provide early detection of outbreaks [21]. The contribution of syndromic surveillance as a tool for local outbreak detection remains a subject open to debate. The strengths of syndromic systems have been reviewed in a study of the current literature and presentation of the views of experts [22]. Syndromic surveillance is currently used for the surveillance of dengue fever within several countries of South America, like in Paraguay. However, for the control of a dengue fever outbreak, biological analyses remain essential for identifying the dengue virus circulating and its serotype, so the appropriate diagnostic capacity must be maintained. It is why biological analysis is the main point of several dengue fever surveillance systems, as in French West Indies [23]. The complementary nature of the two systems was highlighted in French Guiana and has been described by other authors [24], underlining the interest to reinforce timeliness and sensitivity of the system by the introduction of syndromic surveillance. The pertinence of timeliness in public health surveillance systems was also underlined by some authors [25], [26]. The value of syndromic surveillance allowed the military authorities to quickly respond to an outbreak and the civilian authorities to better evaluate the public health situation. This led to negotiations with the national authorities and the French Ministry of Health resulting in improved public health funding to the area.

## Conclusion

The syndromic surveillance allowed an early warning for this outbreak to be issued and a quicker public health response by the armed forces. However, the direct benefit could not be evaluated. The civilian surveillance system has since introduced syndromic surveillance as part of its surveillance strategy. This should enable quicker public health responses in the future.

## Competing interests

The authors declare that they have no competing interests.

## Authors' contributions

J–BM conducted the study and drafted the manuscript. HC developped the 2SE FAG analysis system and its automatization. GT performed the statistical analysis of 2SE FAG data. VA was responsible of the statistical analysis of CVS data. FR was the head of civilian health authorities and conducted the public health response during the outbreak. XD was responsible of the surveillance data analysis within the armed forces, and especially SEA. HJ worked on the evaluation of 2SE FAG and helped to draft the manuscript. PD realized the virological analysis for CVS data. JM was responsible of the virological analysis and took part at the public health response. J–PB was responsible of 2SE FAG program and helped to draft the manuscript. All authors read and approved the final manuscript.

## Pre-publication history

The pre-publication history for this paper can be accessed here:



## Supplementary Material

Additional file 1 «Arrêté du 2 mai 2006 portant création d'un traitement automatisé de données à caractère personnel relatif au suivi des pathologies apparues en mission en Guyane». This text is a part of the Official Journal of the French Republic, giving the authorization to the 2SE FAG project to collect and to analyze some epidemiological data. Click here for file
