# Supplementary material for: Value of syndromic surveillance within the Armed Forces for early warning during a dengue fever outbreak in French Guiana in 2006
Source: BMC Med Inform Decis Mak. 2008 Jul 2;8:29. doi: 10.1186/1472-6947-8-29 (PMC2459153; doi:10.1186/1472-6947-8-29)
Supplement: Additional file 1 — «Arrêté du 2 mai 2006 portant création d'un traitement automatisé de données à caractère personnel relatif au suivi des pathologies apparues en mission en Guyane». This text is a part of the Official Journal of the French Republic, giving the authorization to the 2SE FAG project to collect and to analyze some epidemiological data. [file 1472-6947-8-29-S1.pdf]

Décrets, arrêtés, circulaires

TEXTES GÉNÉRAUX

MINISTÈRE DE LA DÉFENSE

Arrêté du 2 mai 2006 portant création d'un traitement automatisé de données à caractère personnel relatif au suivi des pathologies apparues en mission en Guyane

NOR : *DEFK0600527A*

La ministre de la défense,

Vu la convention du Conseil de l'Europe pour la protection des personnes à l'égard du traitement automatisé des données à caractère personnel, signée à Strasbourg le 28 janvier 1981 ;

Vu la loi n° 78-17 du 6 janvier 1978 relative à l'informatique, aux fichiers et aux libertés, modifiée notamment par la loi n° 2004-801 du 6 août 2004 ;

Vu le décret n° 2005-850 du 27 juillet 2005 relatif aux délégations de signature des membres du Gouvernement ;

Vu le décret n° 2005-1309 du 20 octobre 2005 pris pour l'application de la loi n° 78-17 du 6 janvier 1978 relative à l'informatique, aux fichiers et aux libertés, modifiée par la loi n° 2004-801 du 6 août 2004 ;

Vu le récépissé de la Commission nationale de l'informatique et des libertés en date du 29 mars 2006 portant le numéro 1149659,

Arrête :

Art. 1er. – Il est créé au ministère de la défense, à la direction centrale du service de santé des armées, un traitement automatisé de données à caractère personnel dénommé « 2SE-FAG-(SPE) en Guyane » mis en œuvre par le département d'épidémiologie et de santé publique de l'institut de médecine tropicale et dont les finalités sont le suivi pathologique et géographique des militaires en mission en Guyane.

Art. 2. – Les catégories de données à caractère personnel enregistrées sont celles relatives :

– à l'identité (nom, prénom, sexe, date et lieu de naissance, adresse) ;

– à la vie professionnelle (numéro matricule, grade, unité [d'affectation, élémentaire d'affectation, date d'arrivée, effectif]) ;

– aux missions (identifiant, dates de départ et de retour, nom, unités participant à la mission, effectif, relevé géographique [identifiant de la mission, jour du relevé, latitude, longitude, date d'acquisition GPS, localisation sur carte]) ;

– à la santé (consultation, examens, résultats biologiques, bilan).

Les données à caractère personnel ainsi enregistrées sont conservées cinq ans à compter du départ de l'intéressé de Guyane.

Art. 3. – Les destinataires des données à caractère personnel enregistrées sont, en fonction de leurs attributions respectives et du besoin d'en connaître :

– les médecins d'unité en Guyane ;

– les personnels paramédicaux d'unités en Guyane ;

– les médecins biologistes de l'Institut Pasteur de Cayenne ;

– les médecins du département d'épidémiologie et de santé publique de l'institut de médecine tropicale du service de santé des armées.

Art. 4. – Le droit d'opposition prévu à l'article 38 de la loi du 6 janvier 1978 susvisée ne peut pas être invoqué dans le cadre de ce traitement.

Art. 5. – Le droit d'accès et de rectification prévu aux articles 39 et suivants de la loi précitée s'exerce auprès du département d'épidémiologie et de santé publique de l'institut de médecine tropicale du service de santé des armées, allée du Médecin-Colonel-Jamot, parc du Pharo, BP 46, 13998 Marseille Armées.

Toutefois, lorsque l'exercice du droit d'accès s'applique à des données de santé à caractère personnel, celles-ci peuvent être communiquées à la personne concernée, selon son choix, directement ou par l'intermédiaire d'un médecin qu'elle désigne à cet effet, dans le respect des dispositions de l'article L. 1111-7 du code de la santé publique.

Art. 6. – Le directeur de l'institut de médecine tropicale du service de santé des armées est chargé de l'exécution du présent arrêté, qui sera publié au *Journal officiel* de la République française.

Fait à Paris, le 2 mai 2006.

Pour la ministre et par délégation :
Le directeur central
du service de santé des armées,
B. Lafont
